# Aggravation of collagen-induced arthritis by orally administered *Porphyromonas gingivalis* through modulation of the gut microbiota and gut immune system

**DOI:** 10.1038/s41598-017-07196-7

**Published:** 2017-07-31

**Authors:** Keisuke Sato, Naoki Takahashi, Tamotsu Kato, Yumi Matsuda, Mai Yokoji, Miki Yamada, Takako Nakajima, Naoki Kondo, Naoto Endo, Reiko Yamamoto, Yuichiro Noiri, Hiroshi Ohno, Kazuhisa Yamazaki

**Affiliations:** 1 Research Unit for Oral-Systemic Connection, Division of Oral Science for Health Promotion, Niigata University Graduate School of Medical and Dental Sciences, Niigata, Japan; 2 Division of Periodontology, Niigata University Graduate School of Medical and Dental Sciences, Niigata, Japan; 3 Research Centre for Advanced Oral Science, Niigata University Graduate School of Medical and Dental Sciences, Niigata, Japan; 4 Laboratory for Intestinal Ecosystem, RIKEN Centre for Integrative Medical Sciences (IMS), Yokohama, Japan; 5 Division of Dental Educational Research Development, Niigata University Graduate School of Medical and Dental Sciences, Niigata, Japan; 6 Division of Orthopedic Surgery, Department of Regenerative and Transplant Medicine, Niigata University Graduate School of Medical and Dental Sciences, Niigata, Japan; 7 Institute for Integrated Cell-Materials Science (WPI-iCeMS), Kyoto University, Sakyo, Kyoto Japan; 8 Division of Cariology, Operative Dentistry and Endodontics, Department of Oral Health Science, Niigata University Graduate School of Medical and Dental Sciences, Niigata, Japan; 9 Present Address: Laboratory for Integrative Omics, RIKEN Quantitative Biology Center (QBiC), Osaka, Japan

## Abstract

*Porhyromonas gingivalis*, a causative bacterium of periodontitis, is implicated in the etiology of rheumatoid arthritis (RA), mainly because of expressing peptidyl arginine deiminase (PAD) that generates RA-related autoantigens. However, compared with other periodontopathic bacteria, the precise role of *P*. *gingivalis* in RA is largely unknown. We found that orally administered *P*. *gingivalis* changed the gut microbiome with concomitant elevation of serum endotoxin and inflammatory markers, and impairment of the gut barrier function. Based on findings showing a relationship between gut microbiota and RA, we investigated whether the change of gut microbiota induced by *P*. *gingivalis* and *Prevotella intermedia*, another periodontopathic bacterium without PAD, is associated with collagen-induced arthritis (CIA). DBA/1J mice were orally administered with or without bacteria followed by induction of CIA. *P*. *gingivalis*, but not *P*. *intermedia*, administration significantly aggravated arthritis with increased interleukin-17 levels in sera and culture supernatants, increased Th17 cell proportions among mesenteric lymphocytes, and a significant change in the gut microbiome. However, *P*. *gingivalis* administration did not elevate the level of anti-citrullinated protein antibody. These results suggest a unique role of *P*. *gingivalis* in the link between periodontitis and RA by affecting the gut immune system and the gut microbiota composition.

## Introduction

Periodontal disease is a chronic inflammatory condition of the periodontium induced by periodontopathic bacteria such as *Porphyromonas gingivalis* that destroys tooth-supporting tissue, resulting in tooth loss if left untreated. It is becoming evident that periodontal disease is associated with an increased risk of rheumatoid arthritis (RA) in humans^1, 2^ and animal models^3, 4^. RA is an autoimmune disease characterised by chronic inflammation of the synovial tissues, leading to joint destruction. The majority of RA cases are triggered by an autoimmune response to citrullinated proteins. These proteins are generated by endogenous peptidyl arginine deiminase (PAD) under physiological conditions, but the loss of tolerance in genetically susceptible individuals drives the generation of autoantibodies against citrullinated proteins in the synovia^5, 6^. *P*. *gingivalis* is the only known periodontopathic bacterium that expresses a bacterial PAD^7^. While several studies have demonstrated a positive association between *P*. *gingivalis* infection and anti-cyclic citrullinated protein antibody responses in RA patients^8, 9^, other studies have found no correlation^10, 11^. Furthermore, the hypothesis that PAD links RA with periodontal disease has been disputed^12^.

In animal studies, a comparison of wild-type *P*. *gingivalis* with PAD-deficient *P*. *gingivalis* or *Prevotella intermedia*, which is naturally deficient for PAD, has implicated bacterial PAD as a mechanistic link between periodontal infection with *P*. *gingivalis* and RA^13^. In addition, administration of a Pan-PAD inhibitor, Cl-amidine, reduces the severity of collagen-induced arthritis (CIA)^14^. Conversely, it has been demonstrated that PAD4, which is closely associated with synovial tissue inflammation^15^, is not essential for arthritis development in the K/BxN serum transfer model in C57BL/6 mice^16^. Thus, the role of PAD in the link between periodontitis and RA is not fully elucidated.

In addition to autoimmune responses to citrullinated proteins, proinflammatory cytokines may be another important factor in understanding the association between periodontal disease and RA. Pre-existing extra-synovial inflammation induced by *P*. *gingivalis* is associated with the rapid occurrence in the adjuvant arthritis model^17^. Moreover, it has been demonstrated that *P*. *gingivalis*-induced aggravation of CIA is mediated via increased production of interleukin (IL)-17^18^.

Taken together, *P*. *gingivalis* appears to be involved in the pathogenesis of both periodontal disease and RA. Although the characteristics of *P*. *gingivalis* are worth noting, it is possible that other periodontopathic bacteria or other infections are also involved in RA pathogenesis, suggesting non-specific involvement of bacteria. Nevertheless, the precise mechanisms by which *P*. *gingivalis* infection elevates autoimmune responses are not fully understood.

Recently, gut microbiota has drawn attention as an environmental factor for RA development and progression. Both IL-1 receptor antagonist knockout (IL-1rn^−/−^) and K/BxN mouse models of arthritis remain healthy in germ-free environments. Gavaging these mice with *Lactobacillus* and segmented filamentous bacteria (SFB), respectively, is sufficient for development of autoimmunity and inflammatory arthritis via induction of a robust Th17 response^19, 20^. Most recently, inflammatory arthritis was induced in rats by introducing oral antigens in the setting of mucosal barrier dysfunction^21^. Taken together, these studies suggest a pathogenic role of the gut microbiota in various susceptible animal models and verified a mechanistic relationship between microbiota, mucosal immunity, and joint inflammation.

We have recently shown that oral administration of *P*. *gingivalis* induces endotoxemia, metabolic changes, impairment of the gut barrier function, and dysbiosis of the gut microbiota composition^22, 23^. Considering the close association between periodontitis and RA, the role of gut microbiota in RA pathogenesis and the changes of gut microbiota induced by periodontopathic bacteria, we aimed to clarify whether the association of periodontal disease with RA is due to effects on gut microbiota and gut immune systems exerted by ingested *P*. *gingivalis*. To clarify the specific role of *P*. *gingivalis*, *P*. *intermedia*, another periodontopathic bacterium^24^ that does not express PAD activity, was used as a control.

## Results

### *P*. *gingivalis* but not *P*. *intermedia* administration aggravates CIA

**Figure 1:**
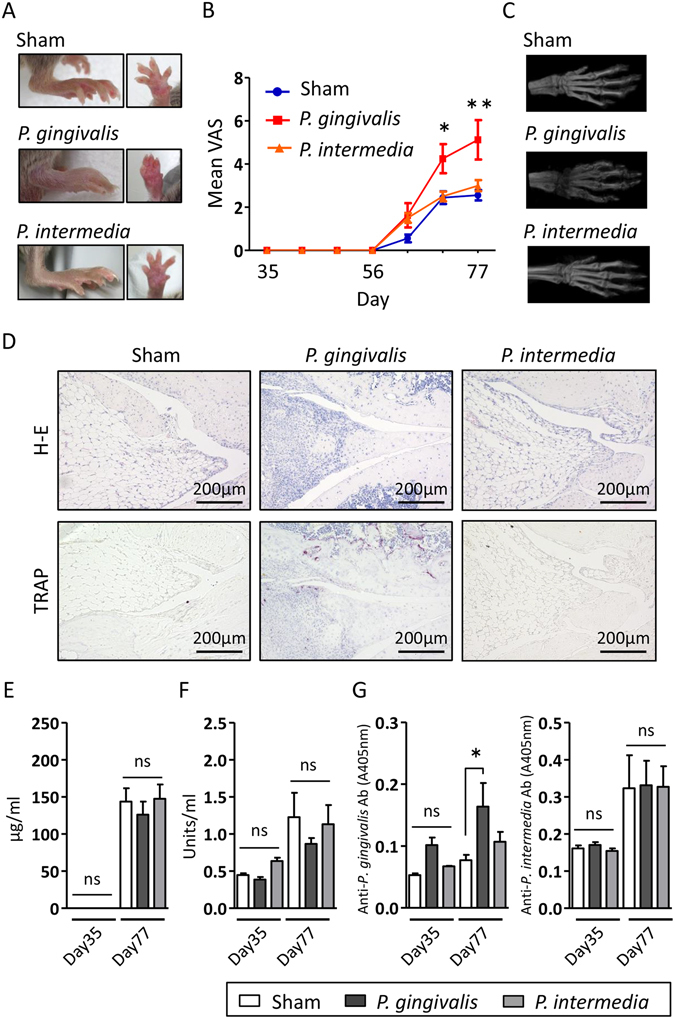
Effect of *P. gingivalis* administration on clinical features and serum levels of antibodies in a CIA model. (**A**) Representative image of the hind paw of sham-, *P. gingivalis*-, and *P. intermedia*-administered mice following CII immunisation. (**B**) Visual assessment score (VAS) of sham-, *P. gingivalis*-, and *P. intermedia*-administered mice. (**C**) Micro-computed tomographic appearance of the paws of sham-, *P. gingivalis*-, and *P. intermedia*-administered mice. (**D**) Histological analysis of the knee joints of sham-, *P. gingivalis*-, and *P. intermedia*-administered mice. Sections were stained with haematoxylin and eosin, and tartrate-resistant acid phosphatase in combination with haematoxylin. Serum levels of anti-CII antibodies (**E**), anti-cyclic citrullinated protein (CCP) antibodies (**F**), and IgG antibodies against *P. gingivalis* W83 and *P. intermedia* ATCC 25611 (**G**) were compared between sham-, *P. gingivalis*-, and *P. intermedia*-administered mice. Sera were obtained immediately before immunisation (day 35) and after induction of CIA (day 77). Data represent the mean ± standard error of the mean (SEM) (N = 6 for (**A**,**B**,**E**,**F** and **G**). *p < 0.05, **p < 0.01, One-way ANOVA with Bonferroni corrections for multiple comparisons.

During the period of infection and primary immunisation, visual assessment scores did not change and demonstrated no difference among *P. gingivalis*-, *P. intermedia*-, and sham-administered groups. After booster immunisation, arthritis was induced in all groups, but the severity of arthritis was significantly higher in the *P. gingivalis*-administered group compared with sham- and *P. intermedia*-administered groups after day 77 (Fig. 1A and B), suggesting that *P. gingivalis*, but not *P. intermedia*, infection affects the disease course rather than the development of arthritis.

These observations were confirmed by micro-computed tomographic findings showing a significantly lower bone mineral density in *P. gingivalis*-administered mice (Fig. 1C).

Histological evaluation of the knee joint after induction of CIA (day 77) demonstrated massive inflammatory cell infiltrates and destruction of the meniscus. Smoothness of the surface structure was lost in the knee cartilage of *P. gingivalis*-administered mice only. In addition, the number of osteoclasts on the inner surface of cartilage was increased in *P. gingivalis*-administered mice compared with *P. intermedia*- and sham-administered mice (Fig. 1D). Taken together, these results suggest that *P. gingivalis* administration specifically accelerates the development of CIA. However, there was no effect of *P. gingivalis* or *P. intermedia* administrations on the anti-CII antibody level (Fig. E). Furthermore, *P. gingivalis* administration did not induce elevation of anti-citrullinated protein antibody (ACPA) production (Fig. 1F) even with elevated levels of serum antibodies against *P. gingivalis* in the *P. gingivalis*-administered mice. No significant antibody production against *P. intermedia* was observed even in the *P. intermedia*-administered mice (Fig. 1G). Therefore, aggravation of arthritis in *P. gingivalis*-administered mice is not attributable to a heightened response to CII by *P. gingivalis* administration or either ACPA production mediated by endogenous PAD or *P. gingivalis*-derived PAD.

### Oral administration of *P. gingivalis* changes the gut microbiota

**Figure 2:**
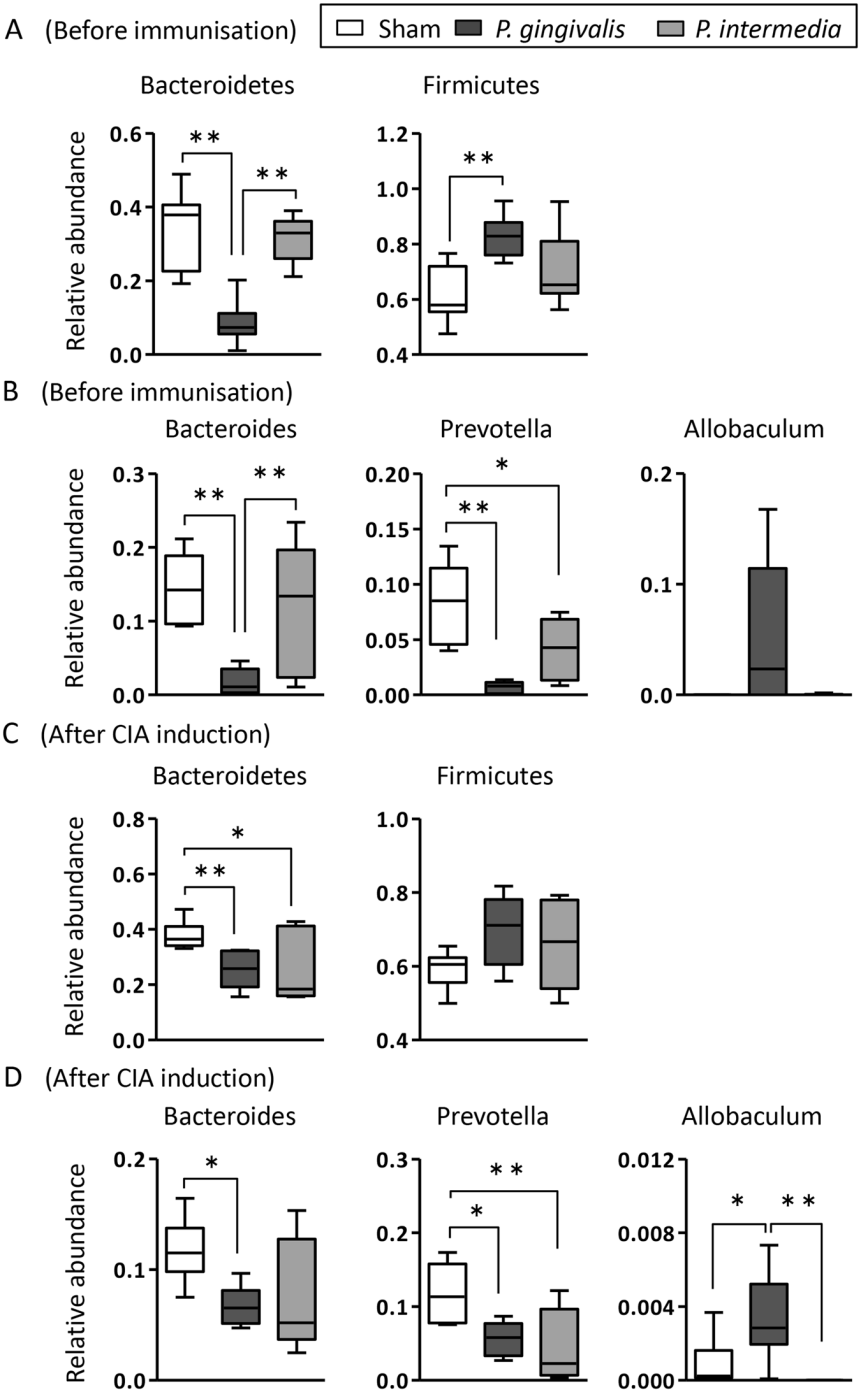
Analysis of the gut microbiota composition in sham-, *P. gingivalis*-, and *P. intermedia*-administered mice by 16S rRNA sequencing. Analyses were carried out using faeces obtained before immunisation (day 35; (**A** and **B**)) and after induction of CIA (day 77; (**C** and **D**)). Relative abundances of each bacterial group at the phylum level before immunisation (day 35; A) and after induction of CIA (day 77; (**C**)) are indicated by box plots. *p < 0.05, **p < 0.01, One-way ANOVA with Bonferroni corrections for multiple comparisons. Box plot showing the relative abundance of the three bacterial genera found in treatment group before immunisation (day 35; **B**) and after induction of CIA (day 77; **D**). *Bacteroides*, *Prevotella*, and *Allobaculum* are represented as percentages on the Y-axis. *p < 0.05, **p < 0.01, One-way ANOVA with Bonferroni corrections for multiple comparisons.

After 5 weeks of bacterial administration, just prior to the initial immunisation (day 35), faeces were analysed for their microbiota composition. The proportions of phylum *Bacteroidetes* was significantly lower in *P. gingivalis*-administered mice compared with either sham-administered or *P. intermedia*-administered mice. Conversely, the proportion of phylum Firmicutes was significantly higher in *P. gingivalis*-administered mice (Fig. 2A), but not in *P. intermedia*-administered mice. No significant difference was observed for other bacterial phyla (data not shown).

At the genus level, the proportions of both *Bacteroides* and *Prevotella* were significantly lower in *P. gingivalis*-administered mice. In *P. interemedia*-administered mice, the proportion of genus *Bacteroides* demonstrated no difference compared with sham-administered mice, but it was significantly higher compared with *P. gingivalis*-administered mice. The proportion of genus *Prevotella* was significantly lower in *P. intermedia*-administered mice compared with sham-administered mice, albeit to a lesser extent than in *P. gingivalis*-administered mice. The proportion of genus *Allobacullum* belonging to phylum Firmicutes tended to be higher only in *P. gingivalis*-administered mice (Fig. 2B), although the differences were not significant.

To analyse the long-term effect of oral administration of *P. gingivalis* on the gut microbiota composition, faeces were obtained after induction of CIA (day 77) and subjected to 16 S rRNA gene analysis. As shown in Fig. 2C, the proportion of phylum *Bacteroides* was significantly lower in *P. gingivalis*- and *P. intermedia*-administered mice, albeit to a lesser extent, compared with sham-administered mice. The proportion of phylum Firmicutes tended to be higher in both *P. gingivalis*- and *P. intermedia*-administered mice compared with sham-administered mice.

The proportions of genuses *Bacteroides* and *Prevotella* were significantly lower in *P. gingivalis*-administered mice compared with sham-administered mice at 6 weeks after termination of the administration. In *P. intermedia*-administered mice, the proportion of genus *Prevotella*, but not genus *Bacteroides*, was significantly lower compared with sham-administered mice. However, similar to the phylum level, the proportion of genus *Bacteroides* tended to be lower compared with sham-administered mice. In addition, the proportion of genus *Allbaculum* tended to be higher only in *P. gingivalis*-administered mice. Thus, the effect of orally administered *P. gingivalis* and *P. intermedia* on the gut microbiota appears to be long lasting (Fig. 2D).

**Figure 3:**
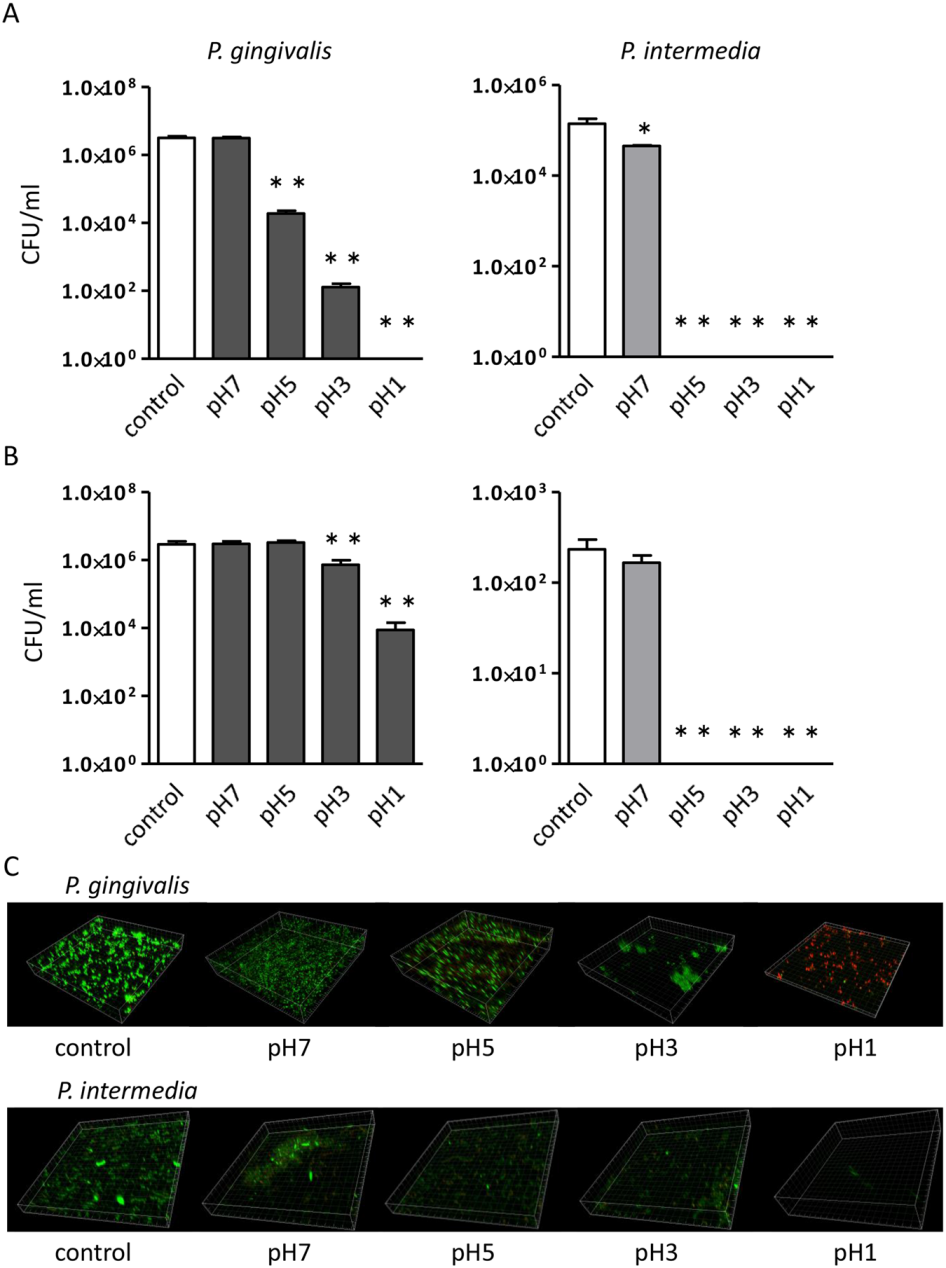
Acid resistance activity of *P. gingivalis* and *P. intermedia* cultures. Colony forming unit (CFU)/ml of planktonic cells (**A**) and biofilm (**B**), and confocal laser scanning microscopy images of biofilm (**C**) after 2 h of treatment with PBS or artificial gastric juice with various pH. Data represent the mean ± SEM (N = 4). *p < 0.05, **p < 0.01, One-way ANOVA with Bonferroni corrections for multiple comparisons.

To analyse the possibility that administered *P. gingivalis* can gain access to the gut through the stomach, *P. gingivalis* was exposed to artificial gastric juice (AGJ). Approximately 1% of *P. gingivalis* planktonic cells were estimated to be viable after 2 h of exposure to AGJ at pH 5 that is equivalent to the pH immediately after a meal (Fig. 3A). The survival rate was dramatically increased by the formation of a *P. gingivalis* biofilm. More than 50% of cells had survived after 2 h of exposure to AGJ even at pH 3 (Fig. 3B and C). Although biofilm formation also increased the acid resistance activity of *P. intermedia* compared with planktonic cells, the survival rate was much lower compared with *P. gingivalis* with complete death at pH 5. Nevertheless, *P. gingivalis* and *P. intermedia* were not detected in faeces obtained either prior to the initial immunisation or at the end of the experimental period irrespective of their acid resistance activity (see Supplementary Fig. S2).

### *P. gingivalis* administration elevates systemic inflammatory mediators and induces Th17 responses in mesenteric lymph nodes, and Peyer’s patches

**Figure 4:**
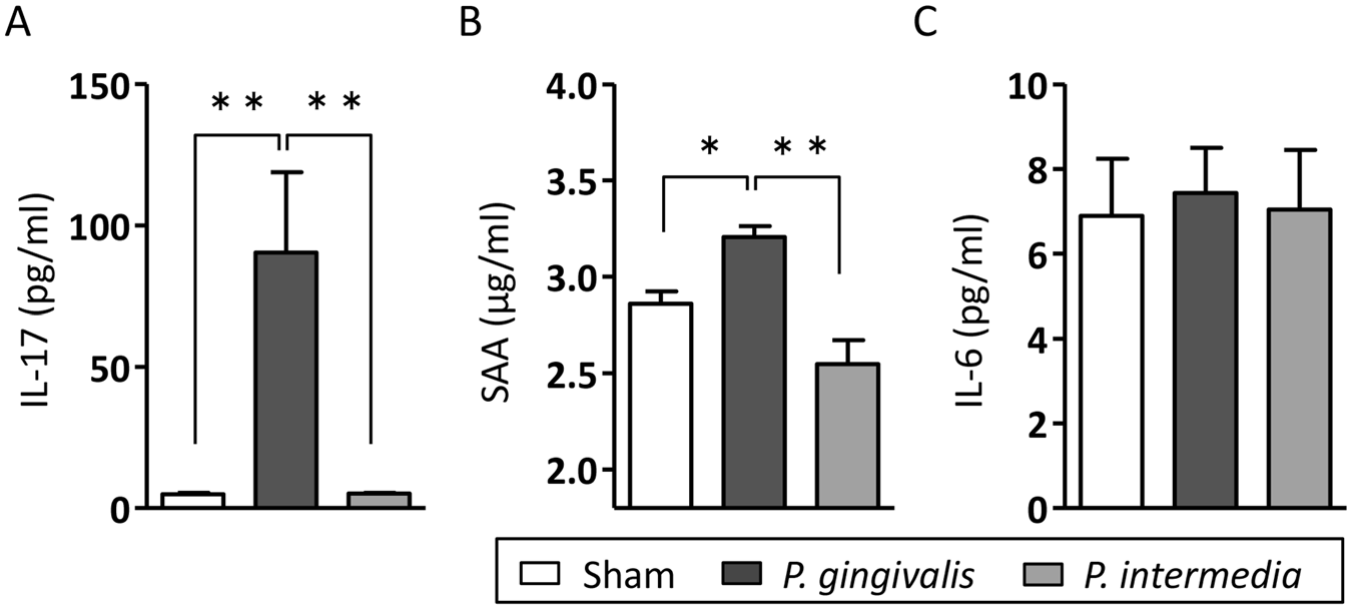
Effect of oral administration of *P. gingivalis* and *P. intermedia* on serum levels of inflammatory markers. Serum levels of IL-17 (**A**), serum amyloid A (**B**), and IL-6 (**C**) were compared between sham-, *P. gingivalis*-, and *P. intermedia*-administered mice. Data are expressed as the mean ± SEM (N = 6). *p < 0.05, **p < 0.01, One-way ANOVA with Bonferroni corrections for multiple comparisons.

The serum level of IL-17 was significantly elevated in *P. gingivalis*-administered mice (Fig. 4A). *P. gingivalis* administration also significantly increased the serum level of amyloid A (SAA; Fig. 4B). No significant increases of IL-17 or SAA in the sera were observed in *P. intermedia*-administered mice compared with sham-administered mice. The serum level of IL-6 was not increased in both *P. gingivalis*- and *P. intermedia*-administered mice (Fig. 4C).

**Figure 5:**
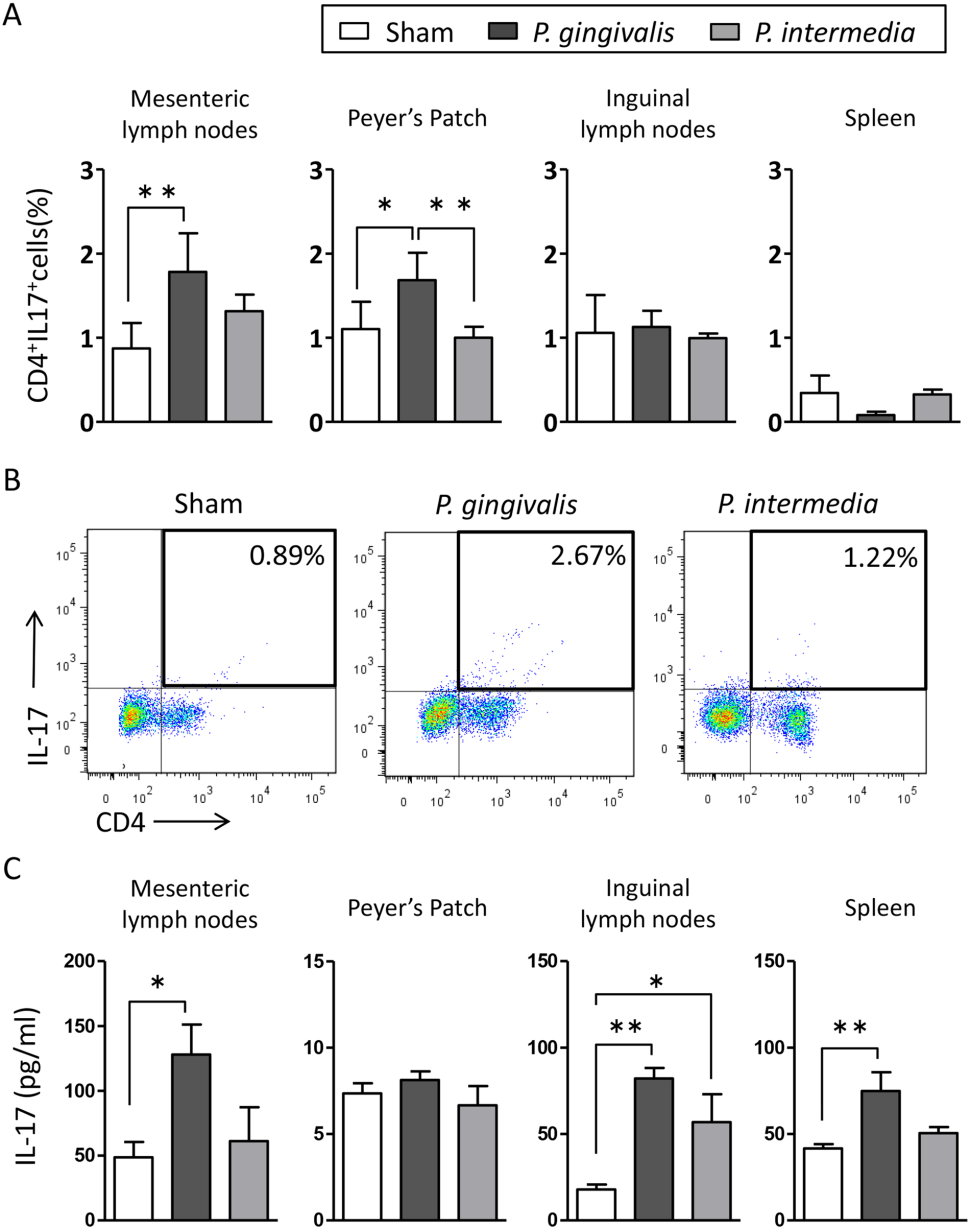
Effects of oral administration of bacteria on the Th17 response of the gut immune system. (**A**) Lymphocyte fractions were obtained from mesenteric lymph nodes, Peyer’s patches, inguinal lymph nodes, and the spleen of sham-, *P. gingivalis*-, and *P. intermedia*-administered mice. Cells were stimulated at a concentration of either 1 × 10^7^/ml (spleen) or 1 × 10^6^/ml (other lymph nodes). The cells were stained with anti-CD4 and anti-IL-17 antibodies, and 1 × 10^4^ cells were analysed by flow cytometry. The percentages of CD4^+^IL-17^+^ cells were compared. Data are expressed as the mean ± SEM (N = 6). *p < 0.05, **p < 0.01, One-way ANOVA with Bonferroni corrections for multiple comparisons. (**B**) Representative plots of one experiment with six mice per group. (**C**) Lymphocytes obtained from the tissues of mice were stimulated with PMA/ionomycin for 24 h. Culture supernatants were analysed by an ELISA. Data represent the mean ± SEM (N = 6). *p < 0.05, **p < 0.01, One-way ANOVA with Bonferroni corrections for multiple comparisons.

To analyse the effect of *P. gingivalis* and *P. intermedia* administrations on the Th17 response, mesenteric and inguinal lymph nodes, Peyer’s patches, and spleens were collected at the end of the experimental period, and the proportions of CD4^+^IL-17^+^ cells were analysed by flow cytometry. The proportions of Th17 cells were significantly elevated in mesenteric lymph nodes and Peyer’s patches, but not in the inguinal lymph nodes or the spleen (Figs 5A and S3) of *P. gingivalis*-administered mice. However, these changes were not observed in *P. intermedia*-administered mice. Representative dot plots of mesenteric lymphocytes are shown in Fig. 5B.

Next, we analysed the level of IL-17 in the culture supernatants of PMA/ionomycin-stimulated lymphocytes from mesenteric lymph nodes, Peyer’s patches, inguinal lymph nodes, and spleens by an enzyme-linked immunosorbent assay (ELISA). Cells from the mesenteric lymph nodes and spleens of *P. gingivalis*-administered mice, but not *P. intermedia*-administered mice, produced significantly higher amounts of IL-17 compared with those from sham-administered mice. In *P. intermedia*-administered mice, only cells from inguinal lymph nodes produced a significantly higher amount of IL-17 compared with sham-administered mice, but lower than those from *P. gingivalis*-administered mice (Fig. 5C).

### *P. gingivalis* administration upregulates the expression of proinflammatory genes in the gut

**Figure 6:**
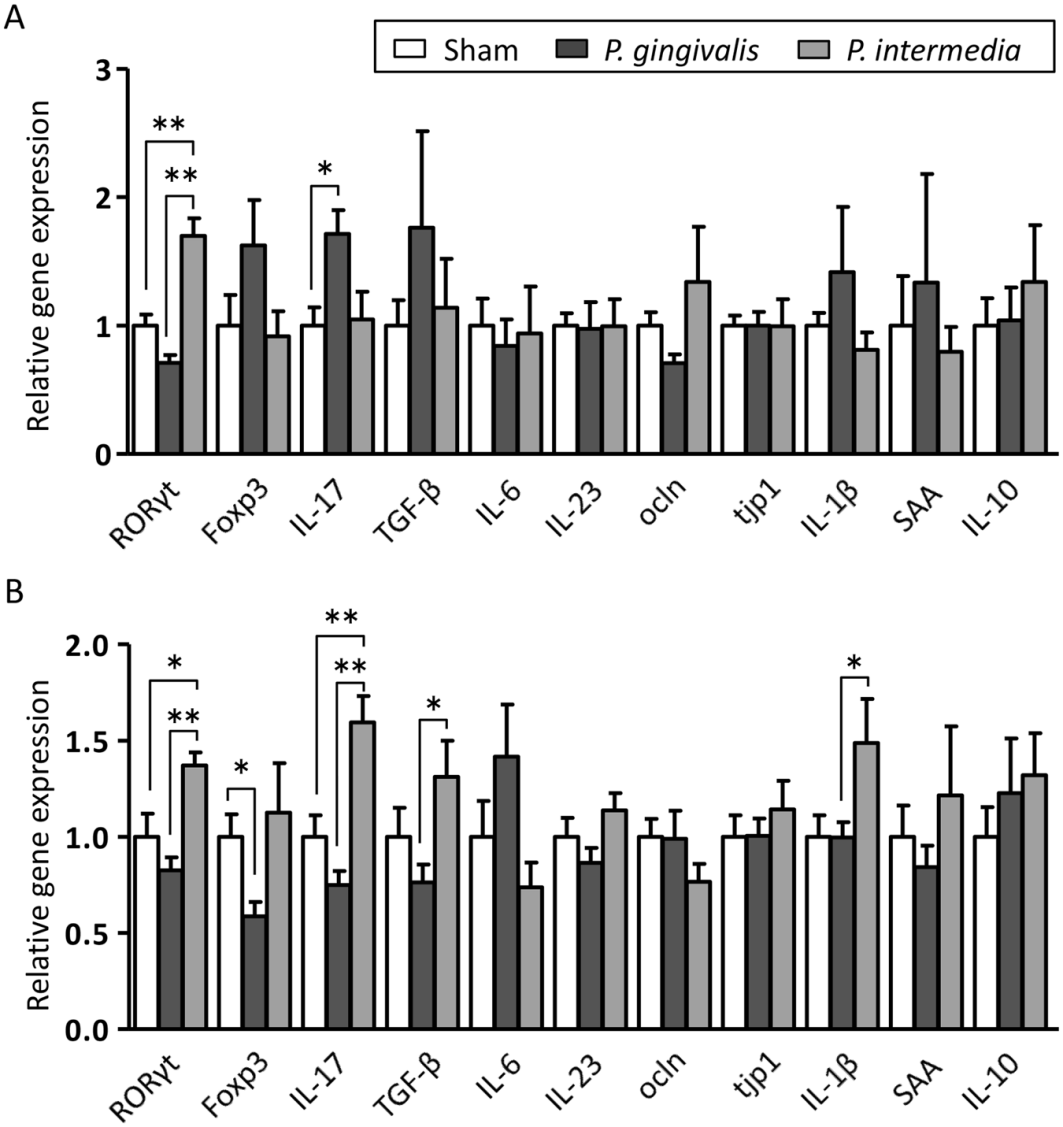
Effect of *P. gingivalis* and *P. intermedia* administration on gene expression of the gut. (**A**) Relative gene expression levels in the small intestines of sham-, *P. gingivalis*-, and *P. intermedia*-administered mice (N = 7 in each group). (**B**) Relative gene expression levels in the large intestines of sham-, *P. gingivalis*-, and *P. intermedia*-administered mice (N = 7 in each group). Relative mRNA expression levels of genes were normalised to the relative quantity of glyceraldehyde-3-phosphate dehydrogenase mRNA. Data represent the mean ± SEM (N = 7). *p < 0.05, **p < 0.01, One-way ANOVA with Bonferroni corrections for multiple comparisons.

Significant upregulation of IL-17 gene expression was observed in the ileum of *P. gingivalis*-administered mice (Fig. 6A). Conversely, significant upregulation of *Rorγt* was observed in the ileum of *P. intetermedia*-administered mice. The expression of *Foxp3* and *Tgfb* tended to be higher, whereas the expression of *Rorγt* and *Ocln* tended to be lower in the ileum of *P. gingivalis*-administered mice compared with sham-administered mice (Fig. 6A). However, they did not reach statistical significance.

In the colon, the expression *Foxp3* was significantly downregulated in *P. gingivalis*-administered mice, whereas the expression of *Rorγt* and *Il17* was significantly upregulated in *P. intermedia*-administered mice. The expression of *Tgfb* and *Il1b* was significantly higher in *P. intermedia*-administered mice compared with *P. gingivalis*-administered mice. A trend towards upregulation of expression was observed for *IL6* in *P. gingivalis*-administered mice (Fig. 6B).

## Discussion

To gain an insight into the causal mechanism of RA by periodontal infection, we used a mouse experimental arthritis model. Although the method to induce periodontitis is variable, we employed oral administration of bacteria to mimic the condition of periodontal disease in the present study because this model better reflects the conditions in periodontitis patients, who swallow periodontopathic bacteria with their saliva, than the other model in which *P. gingivalis* is injected subcutaneously^13, 17^. In addition, the bacteria used for infection are also variable^3, 4, 13, 17, 18, 25–27^. However, much of the attention has been focused on the relevance of *P. gingivalis* to the etiology of RA. We confirmed that orally administered *P. gingivalis* W83 aggravated CIA in DBA/1 mice. It is noteworthy that *P. intermedia*, another periodontopathic bacterium, had no effect on the severity or progression of CIA.

ACPA is not only important for diagnosis but also pathogenesis because the antibodies drive autoimmunity in RA^28, 29^. In this respect, *P. gingivalis* has drawn attention primarily because it is the only oral bacteria that expresses PAD (PPAD) that is involved in the generation of citrullinated proteins^9^. In this respect, *P. intermedia*, another periodontopathic bacterium that does not express PAD, was used as a control to clarify that PPAD was in fact involved in the aggravation of arthritis in the present study.

The role of PAD in the pathogenesis of RA in relation to periodontitis is controversial. Several studies have demonstrated an association of periodontitis with ACPA^8^, elevated levels of antibodies against citrullinated PPAD in RA patients^9^, and elevated PAD and PPAD activities within the periodontium of RA and non-RA patients with periodontitis^30^. Conversely, other studies have demonstrated no association of anti-*P. gingivalis* antibodies with RA or the ACPA status, no association of *P. gingivalis* with ACPA^2^, and no correlation of antibodies against PPAD with ACPA or disease activity of RA^12^. A more recent study demonstrated that *Aggregatibacter actinomycetemcomitans*, another periodontopathic bacterium, but not *P. gingivalis* or *P. intermedia*, is associated with RA through leukotoxin A-mediated hypercitrullination^31^. Our study demonstrated that administration of neither *P. gingivalis* nor *P. intermedia* induced ACPA. Therefore, we conclude that PPAD is not involved in the acceleration of arthritis development at least in our CIA model.

Generation of anti-CII antibodies is essential for the development of CIA, and the level of anti-CII antibodies is associated with the severity of arthritis. Because immunisation with CII induced equal levels of anti-CII antibodies among the groups, either *P. gingivalis* or *P. intermedia* administrations are not involved in the anti-CII antibody-mediated aggravation of arthritis.

The role of IL-17 and its producer, Th17 cells, has been of particular interest in RA because neutralising antibodies against IL-17 have shown promise for the treatment of RA^32^. The major role of IL-17 has also been demonstrated in the CIA model using IL-17-deficient mice^33^.

Our study demonstrated a significantly higher level of serum IL-17 in *P. gingivalis*-administered mice compared with that in *P. intermedia*- and sham-administered mice. This increase was induced by *P. gingivalis* administration but not immunisation with CII as mentioned above.

The proportion of CD4^+^IL-17^+^ cells in the spleen was not significantly different between either *P. gingivalis*- and sham-administered mice or *P. intermedia*- and sham-administered mice. However, IL-17 production was significantly upregulated upon stimulation only in cells from *P. gingivalis*-administered mice, indicating the presence of activated Th17 cells in the spleen of *P. gingivalis*-administered mice. Interestingly, the proportion of Th17 cells was increased in both mesenteric lymph nodes and Peyer’s patches of *P. gingivalis*-administered mice, and cells were activated in the mesenteric lymph nodes. It is becoming well recognised that the profile of the gut immune system is affected by gut microbiota and vice versa. Furthermore, the interaction between gut microbiota and the gut immune system plays a pivotal role in health and disease^34^. Wu *et al*. demonstrated that mono-colonisation of SFB in the gut induces differentiation of Th17 cells, resulting in development of arthritis in genetically susceptible mice^20^. Therefore, we speculated that oral administration of *P. gingivalis* increases the proportion of SFB in the gut. However, we did not detect SFB in faecal or gut tissue samples by PCR using specific primers (Supplementary Fig. S2A). Although SFB are well-known bacteria involved in the induction of Th17 cells, it has been shown that other bacteria induce Th17 cells, such as *Citrobacter rodentium*, *Escherichia coli* O157^35^, and altered Schaedler flora^36^. Because *P. gingivalis* is also associated with enhanced Th17 responses^37^, we examined the establishment of *P. gingivalis* in the gut but did not detect them (Supplementary Fig. S2B). This lack of detection may not be due to acidic degradation by gastric fluid because *P. gingivalis* show an acid-resistant activity. In contrast to *P. gingivalis*, the acid-resistant activity of *P. intermedia* was weak.

Dysbiosis of the gut microbiota is associated with aberrant immune responses in human RA and mouse arthritis models. Furthermore, RA patients demonstrate disturbances of gut microbiota and alterations of oral microbiota compared with healthy subjects. Interestingly, both the gut microbiome and oral microbiome resolve partially after RA treatment^38^. These findings suggest that oral and gut microbiomes are at least in part mutually associated. In this connection, we have previously demonstrated that repeated oral administration of *P. gingivalis* induces an alteration of the gut microbiome together with inflammatory changes in the adipose tissues and liver of C57BL/6 mice^22^. The effect of oral bacteria on the gut microbiota has recently been shown in humans^39^. The study revealed a major change in the gut microbiota of patients with liver cirrhosis occurring because of massive invasion of the gut by oral bacterial species^39^. In contrast to these studies, it has been demonstrated that oral administration of *P. gingivalis* induces periodontal microbiota dysbiosis and insulin resistance without alteration of the gut microbiota composition in mice. However, these changes are not observed in *P. gingivalis*-infected normal chow-fed mice^40^. Because the effect of a high fat diet on the gut microbiota appears to be greater compared with swallowed *P. gingivalis*, the difference in the results could be attributable to the difference in diet.

In the present study, orally administered *P. gingivalis* also perturbed gut microbiomes in DBA/1 mice and affected subsequent induction of CIA. This pattern of change persisted even after termination of *P. gingivalis* administration until the end of the experimental period. These changes of gut microbiomes were also associated with the changes in gene expression of the gut. The pattern of the changes in gut microbiota seen in this study was different from that in our previous study^22, 23^. Although precise reasons for the difference are unknown, the difference in the mouse strain may explain the contrasting results at the phylum level. *P. intermedia* administration also induced a change in the gut microbiota composition to some extent despite having no effect on CIA pathology.

Identification of pathology-related bacteria in RA is ongoing and no consistent results have been reported. Scher *et al*. found an increase of *Prevotella copri* in the gut microbiota of patients with new-onset untreated RA^41^. Although the authors identified unique *Prevotella* genes that correlated with the disease and colonisation of *P. copri* resulting in an increase of sensitivity to chemically induced colitis, direct evidence of *P. copri* in RA pathology is still lacking. We found a reduction in genus *Prevotella* after *P. gingivalis* administration. However, it is unknown whether bacteria belonging to genus *Prevotella* have similar characteristics to those of *P. copri*. We also found a reduction of genus *Bacteroides* in *P. gingivalis*-administered mice. In this regard, Vaahtovuo *et al*. revealed lower levels of the *Bacteroides*-*Porphyromonas*-*Prevotella* bacterial group in patients with early RA compared with non-RA controls^42^. It is worth noting that the observed change in the relative abundance of Firmicutes and Bacteroidetes in the faecal samples induced by *P. gingivalis* administration resembles the phylum-level population shifts reported as high fat diet-induced alterations in a mouse model^43^.

In contrast to genuses *Bacteroides* and *Paraprevotella*, the proportion of the minor population genus *Allobaculum* tended to be higher in *P. gingivalis*-administered mice. The role of minor populations of gut bacteria, such as *Allobaculum*, in RA pathology is still elusive. Recently, Chen *et al*. showed that a rare bacterial lineage (*Collinsella*) associated with downregulation of ZO-1 and Occludin, which regulate gut permeability, expands in the intestines of RA patients. It was also shown that *Collincella* have a proinflammatory effect on Caco-2 cells by stimulating production of IL-17A^44^. In the present study, gene expression of occludin tended to be decreased and that of IL-17 was significantly increased in the ileum of *P. gingivalis*-administered mice at 6 weeks after termination of *P. gingivalis* administration. These findings led us to speculate that lipopolysaccharides from gut bacteria readily enter systemic circulation and are capable of potentiating CIA^45^. In fact, oral administration of *P. gingivalis* is reported to decrease the gut barrier function as evidenced by reduced expression of tight junction genes^22, 23^ and elevated endotoxin levels in systemic circulation.

Collectively, our results provide another possible mechanism for the link between periodontitis and RA, whereby the unique periodontopathic bacterium *P. gingivalis* induces the change of gut microbiota and affects the gut immune system. This suggests that *P. gingivalis*-induced gut microbiota composition shifts the gut immune profile towards Th17 dominance. However, the specific bacteria involved in the Th17 cell differentiation and activation in the gut in our study are yet to be determined. In addition, we have not examined whether suppression of the effect of *P. gingivalis* can alter the course or outcome of CIA. Further studies aiming at clarifying the above points will contribute to a much better understanding of the mechanism linking periodontitis and RA in terms of the gut microbiota.

## Materials and Methods

### Mice

Six-week-old male DBA/1J mice were obtained from Japan SLC. The mice were acclimatised under specific pathogen-free conditions and fed regular chow and sterile water until the commencement of infection at 7 weeks of age. All animal experiments were conducted in accordance with the Regulations and Guidelines on Scientific and Ethical Care and Use of Laboratory Animals of the Science Council of Japan, enforced on June 1, 2006. The study protocol was approved by the Animal Care and Use Committee at Niigata University (Permit Number: 195-2).

### Bacterial infection

*P. gingivalis* strain W83 and *P. intermedia* strain ATCC 25611 maintained in our laboratory were used in the present study. A total of 1 × 10^9^ colony-forming units (CFUs) of each live bacterium suspended in 100 µl PBS with 2% carboxymethyl cellulose (Sigma-Aldrich, St. Louis, MO) was administered to the oral cavity of each mouse through a feeding needle twice a week for 5 weeks. The number of administered bacteria was determined by considering body weight and the number of bacteria in the saliva of periodontitis patients. The control group was sham administered 100 µl PBS with 2% carboxymethyl cellulose without bacteria. After administration, all mice were allowed to eat and drink ad libitum.

### Arthritis induction and evaluation

The next day after the final administration of bacteria or the vehicle only, all mice were immunised with CII (Chondrex, Inc., Redmond, WA) emulsified in complete Freund’s adjuvant (Chondrex) and received a booster immunisation with CII in incomplete adjuvant (Chondrex) at 3 weeks after primary immunisation (see Supplementary Fig. S1).

Arthritis was scored by a blinded examiner using a visual assessment scoring (VAS) system with a scale of 0–4 per limb as described previously^46^. Arthritis was also assessed by evaluation of bone destruction by micro-computed tomography (Skyscan 1174; Skyscan, Kontich, Belgium). A region of interest was set around all bones in the whole scanned image. Bone erosion within the hind paws was analysed by CTvol ver. 1.9.4 (SkyScan).

For histological analyses, forepaws were fixed in 4% (vol/vol) paraformaldehyde, decalcified with EDTA, and embedded in paraffin. Ankle joint sections (5 µm thick) in the sagittal direction along the long axis of the hind paw were stained with haematoxylin and eosin. The cells were imaged by microscopy (Biozero BZ-X700; Keyence, Tokyo, Japan).

The sections were also stained for tartrate-resistant acid phosphatase using an osteoclast detection kit (Primary Cell Co., Ltd., Sapporo, Japan) and counterstained with haematoxylin.

### Analysis of sera

Blood was obtained from the mouse orbital sinus, and sera were stored at −20 °C until use. The amounts of antibodies against CII-specific immunoglobulins in the serum were measured using an ELISA kit (Chondrex) for quantitation of mouse IgG. Levels of ACPAs were measured using a commercially available ELISA kit (Euro-Diagnostica, Malmö, Sweden). For this experiment, alkaline phosphatase-conjugated goat anti-mouse IgG (Abcam, Cambridge, UK) was used as the secondary antibody instead of the secondary antibody included in the kit. Absorbance at 405 nm was measured with an ELISA microplate reader (Molecular Devices, Sunnyvale CA). The level of IL-17, serum amyloid A (SAA), and IL-6 in sera was determined using a commercially available ELISA kit (BioSource, Camarillo, CA for IL-17; Tridelta Development, Maynooth, Ireland for SAA; Thermo Fisher Scientific, Waltham, MA for IL-6) according to the manufacturers’ instructions. The lower detection limit of IL-17, SAA, and IL-6 by the ELISA kits was 4 pg/ml, 0.03 μg/ml, and 0.2 pg/ml, respectively. Antibody responses to antigens of periodontopathic bacteria were assessed by ELISA and evaluated according to the method as described previously^47^.

### Flow cytometry and cytokine production of lymphocytes

To analyse intracellular expression of IL-17, cells from each lymphoid tissue were adjusted to a concentration of 1 × 10^6^ in RPMI-1640 (Sigma-Aldrich, St. Louis, MO) supplemented with 10% FBS (Sigma-Aldrich) and then stimulated with phorbol 12-myristate 13-acetate (PMA, 50 ng/ml; Sigma-Aldrich) and ionomycin (1 µg/ml; Calbiochem, San Diego, CA) in the same medium for 24 h. BD Golgi Plug™ (BD Biosciences, San Jose, CA) was added at 16 h after the start of the incubation. After harvesting the cells by centrifugation, a BD Cytofix/Cytoperm Plus Fixation/Permeabilization Kit (BD Biosciences) was used for staining with a FITC-labelled antibody against IL-17A and a PerCP-labelled antibody specific for CD4 (both from eBioscience, San Diego, CA) according to the manufacturer’s instructions. The expression level of each molecule was analysed using a FACS Aria II and FLOWJO (TOMY Digital Biology, Tokyo, Japan). The resultant supernatants were subjected for IL-17A protein assay by ELISA (eBioscience).

### DNA extraction

Bacterial DNA was extracted from faeces as described previously^48^. In brief, faeces were collected before commencement of immunisation (day 35) and at the end of experiments (day 77). Freeze-dried faeces were suspended in buffer (10% sodium dodecyl sulfate, 10 mM Tris-HCl, and 1 mM EDTA, pH 8.0). Faeces in the buffer were disrupted with 0.1 mm zirconia/silica beads (BioSpec Products, Inc., Bartlesville, OK) by shaking at 1500 rpm for 10 min using a ShakeMaster (Hirata Co., Tokyo, Japan). After shaking, bacterial DNA was purified using a phenol/chloroform/isoamyl alcohol (25:24:1) solution. The DNA was precipitated by addition of ethanol and sodium acetate. RNase treatment and polyethylene glycol precipitation were then performed.

### Determination of gut microbiota by deep sequencing

The V4 variable region (515F–806R) of the 16S rRNA genes of bacteria was sequenced on an Illumina Miseq, following the method of Kozich *et al*.^49^. Each reaction mixture contained 15 pmol of each primer, 0.2 mM deoxyribonucleoside triphosphates, 5 µl of 10× Ex Taq HS buffer, 1.25 U Ex Taq HS polymerase (Takara Bio, Inc., Shiga, Japan), 50 ng extracted DNA, and sterilised water to reach a final volume of 50 μl. PCR conditions were as follows: 95 °C for 2 min and then 25 cycles of 95 °C for 20 s, 55 °C for 15 s, and 72 °C for 5 min, followed by 72 °C for 10 min.

The PCR product was purified by AMPure XP (Beckman Coulter, Inc., Brea, CA) and quantified using a Quant-iT PicoGreen ds DNA Assay Kit (Life Technologies Japan Ltd., Tokyo, Japan). Mixed samples were prepared by pooling approximately equal amounts of PCR amplicons from each sample. The pooled library was analysed with an Agilent High Sensitivity DNA Kit on an Agilent 2100 Bioanalyzer (Agilent Technologies, Santa Clara, CA). Real-time PCR for quantification was performed on the pooled library using a KAPA Library Quantification Kit for Illumina following the manufacturer’s protocols.

Based on the quantification, the sample library was denatured and diluted. A sample library with a 20% denatured PhiX spike-in was sequenced by Miseq using a 500 cycles kit. We obtained 2 × 250 bp paired-end reads.

Taxonomic assignments and estimation of relative abundance of sequencing data were performed using the analysis pipeline of the QIIME software package^50^. An operational taxonomic unit (OTU) was defined at 97% similarity. OTUs indicating relative abundance of under 0.05% were filtered to remove noise. The OTU was assigned a taxonomy based on a comparison with the Silva database using UCLUST^51, 52^.

### Acid-resistance activity of *P. gingivalis* and *P. intermedia*

AGJ was prepared at the time of use, referring to the method described by Takahashi *et al*.^53^ Pepsin (2.0 g/l; Sigma-Aldrich) and 3.5 g/l sodium chloride (Wako Pure Chemical Industries, Osaka, Japan) were dissolved in ultrapure water, and the solution was adjusted to pH 1, 3, 5, or 7 with HCl or NaOH. Following the pH adjustment, AGJ was sterilised using a syringe filter with a pore size of 0.2 µm (Millex-FG Filter Unit, SLFG J25 LS; Millipore, Billerica, MA).

Freshly prepared AGJ was dispensed into a 6-well plate (Corning, New York, NY), and bacterial cells were added at a final concentration of 5 × 10^6^ CFU/ml. As a control, bacterial cells were inoculated into PBS. Cells were incubated for 2 h at 37 °C under microaerobic conditions using an AnaeroPack®-MicroAero (Mitsubishi Gas Chemical Co. Inc., Tokyo, Japan). To count colonies, the AGJ-treated cells were inoculated onto blood agar plates.

We further analysed the acid-resistant activity of biofilm-formed bacteria.

Details of the bacterial biofilm formation model using a modified Robbins device (MRD) and hydroxyapatite (HA) disks have been described previously^54^.

After biofilm formation, the biofilm samples on the HA disks obtained from the MRD were washed with PBS. The biofilm cells were scraped from the HA disks and resuspended in AGJ dispensed into a 6-well plate (Corning). As a control, biofilm cells were inoculated into PBS. AGJ treatment and plating for colony counting were performed as described above.

The biofilm from the MRD was stained using a Live/Dead® BacLight™ Bacterial Viability Kit (L7007; Molecular Probes, Eugene, OR) and observed by confocal laser scanning microscopy (LSM-700; Carl Zeiss, München-Hallbergmoos, Germany) as described previously^55^.

### Analysis of gene expression in the intestines

Total RNA from the small and large intestines collected at 24 h after final administration of bacteria or vehicle only was extracted using TRI Reagent® (Molecular Research Center, Cincinnati, OH). cDNA was synthesised with Transcriptor Universal cDNA Master (Roche). Primers and probes for real-time PCR were purchased from Life Technologies. Reactions were carried out in a final volume of 25 µl in a LightCycler® 96 System (Roche) using TaqMan Gene Expression Assays (Life Technologies) containing 900 nM of each primer and 250 nM probe. The reactions consisted of a 10-min incubation at 95 °C, followed by 45 cycles of a 2-step amplification procedure including annealing/extension at 60 °C for 1 min and denaturation for 15 s at 95 °C. LightCycler^®^ 96 software (Roche) was used to analyse the standards and carry out the quantification. The relative quantity of each mRNA was normalised to the relative quantity of glyceraldehyde-3-phosphate dehydrogenase mRNA.

### Statistical analysis

The number of mice in each group was based on previous experiments investigating the effect of oral administration of *P. gingivalis* on gut microbiota and the gene expression profile in various tissues. The data were tested for normality using the Kolmogorov-Smirnov test. Because most, but not all, data sets showed a parametric distribution, all data were assessed by one-way ANOVA with Bonferroni corrections for multiple comparisons using GraphPad Prism® (GraphPad Software, Inc., La Jolla, CA). Analysis of similarity (ANOSIM) was performed to test differences in the bacterial community compositions among groups (e.g., *P. gingivalis*-, *P. intermedia*-, and sham-administered samples) using the vegan package in R (http://cran.at.r-project.org/). It calculates R as a statistical value that describes the level of similarity between each pair in ANOSIM. Values close to unity indicate completely different communities in two groups, whereas a zero value indicates a complete overlap or similarity (null hypothesis). A probability value of p < 0.05 was considered as statistically significant.

### Data availability

The data sets generated and/or analysed during the present study are either included in the manuscript or available from the corresponding author on reasonable request.

## Electronic supplementary material

Supplementary information

## References

[1] Arvikar SL (2013). Clinical correlations with Porphyromonas gingivalis antibody responses in patients with early rheumatoid arthritis. Arthritis Res Ther.

[2] Scher JU (2012). Periodontal disease and the oral microbiota in new-onset rheumatoid arthritis. Arthritis Rheum.

[3] Marchesan JT (2013). Porphyromonas gingivalis oral infection exacerbates the development and severity of collagen-induced arthritis. Arthritis Res Ther.

[4] Cantley MD, Haynes DR, Marino V, Bartold PM (2011). Pre-existing periodontitis exacerbates experimental arthritis in a mouse model. J Clin Periodontol.

[5] Klareskog L, Catrina AI, Paget S (2009). Rheumatoid arthritis. Lancet.

[6] Klareskog L, Ronnelid J, Lundberg K, Padyukov L, Alfredsson L (2008). Immunity to citrullinated proteins in rheumatoid arthritis. Annu Rev Immunol.

[7] McGraw WT, Potempa J, Farley D, Travis J (1999). Purification, characterization, and sequence analysis of a potential virulence factor from Porphyromonas gingivalis, peptidylarginine deiminase. Infect Immun.

[8] Mikuls TR (2014). Periodontitis and Porphyromonas gingivalis in patients with rheumatoid arthritis. Arthritis Rheumatol.

[9] Quirke AM (2014). Heightened immune response to autocitrullinated Porphyromonas gingivalis peptidylarginine deiminase: a potential mechanism for breaching immunologic tolerance in rheumatoid arthritis. Ann Rheum Dis.

[10] Reichert S (2015). Association of levels of antibodies against citrullinated cyclic peptides and citrullinated a-enolase in chronic and aggressive periodontitis as a risk factor of Rheumatoid arthritis: a case control study. J Transl Med.

[11] Seror R (2015). Association of anti-Porphyromonas gingivalis antibody titers with nonsmoking status in early rheumatoid arthritis: Results from the prospective French cohort of patients with early rheumatoid arthritis. Arthritis Rheumatol.

[12] Konig MF, Paracha AS, Moni M, Bingham CO, Andrade F (2015). Defining the role of Porphyromonas gingivalis peptidylarginine deiminase (PPAD) in rheumatoid arthritis through the study of PPAD biology. Ann Rheum Dis.

[13] Maresz KJ (2013). Porphyromonas gingivalis facilitates the development and progression of destructive arthritis through its unique bacterial peptidylarginine deiminase (PAD). PLoS Pathog.

[14] Willis VC (2011). N-a-benzoyl-N5-(2-chloro-1-iminoethyl)-L-ornithine amide, a protein arginine deiminase inhibitor, reduces the severity of murine collagen-induced arthritis. J Immunol.

[15] Foulquier C (2007). Peptidyl arginine deiminase type 2 (PAD-2) and PAD-4 but not PAD-1, PAD-3, and PAD-6 are expressed in rheumatoid arthritis synovium in close association with tissue inflammation. Arthritis Rheum.

[16] Rohrbach AS, Hemmers S, Arandjelovic S, Corr M, Mowen KA (2012). PAD4 is not essential for disease in the K/BxN murine autoantibody-mediated model of arthritis. Arthritis Res Ther.

[17] Bartold PM, Marino V, Cantley M, Haynes DR (2010). Effect of Porphyromonas gingivalis-induced inflammation on the development of rheumatoid arthritis. J Clin Periodontol.

[18] de Aquino SG (2014). Periodontal pathogens directly promote autoimmune experimental arthritis by inducing a TLR2- and IL-1-driven Th17 response. J Immunol.

[19] Abdollahi-Roodsaz S (2008). Stimulation of TLR2 and TLR4 differentially skews the balance of T cells in a mouse model of arthritis. J Clin Invest.

[20] Wu HJ (2010). Gut-residing segmented filamentous bacteria drive autoimmune arthritis via T helper 17 cells. Immunity.

[21] Wu D (2013). Oral antigens induce rheumatoid arthritis-like inflammation in a rat model. Inflamm Res.

[22] Arimatsu K (2014). Oral pathobiont induces systemic inflammation and metabolic changes associated with alteration of gut microbiota. Sci Rep.

[23] Nakajima M (2015). Oral administration of *P. gingivalis* induces dysbiosis of gut microbiota and impaired barrier function leading to dissemination of enterobacteria to the liver. PLoS One.

[24] Wegner N (2010). Peptidylarginine deiminase from Porphyromonas gingivalis citrullinates human fibrinogen and alpha-enolase: implications for autoimmunity in rheumatoid arthritis. Arthritis Rheum.

[25] Gully N (2014). Porphyromonas gingivalis peptidylarginine deiminase, a key contributor in the pathogenesis of experimental periodontal disease and experimental arthritis. PLoS One.

[26] Queiroz-Junior CM (2012). Experimental arthritis exacerbates Aggregatibacter actinomycetemcomitans-induced periodontitis in mice. J Clin Periodontol.

[27] Trombone AP (2010). Periodontitis and arthritis interaction in mice involves a shared hyper-inflammatory genotype and functional immunological interferences. Genes Immun.

[28] Schellekens GA, de Jong BA, van den Hoogen FH, van de Putte LB, van Venrooij WJ (1998). Citrulline is an essential constituent of antigenic determinants recognized by rheumatoid arthritis-specific autoantibodies. J Clin Invest.

[29] van de Stadt LA (2011). Development of the anti-citrullinated protein antibody repertoire prior to the onset of rheumatoid arthritis. Arthritis Rheum.

[30] Laugisch O (2016). Citrullination in the periodontium-a possible link between periodontitis and rheumatoid arthritis. Clin Oral Investig.

[31] Konig MF (2016). Aggregatibacter actinomycetemcomitans-induced hypercitrullination links periodontal infection to autoimmunity in rheumatoid arthritis. Sci Transl Med.

[32] Genovese MC (2010). LY2439821, a humanized anti-interleukin-17 monoclonal antibody, in the treatment of patients with rheumatoid arthritis: A phase I randomized, double-blind, placebo-controlled, proof-of-concept study. Arthritis Rheum.

[33] Nakae S, Nambu A, Sudo K, Iwakura Y (2003). Suppression of immune induction of collagen-induced arthritis in IL-17-deficient mice. J Immunol.

[34] Honda K, Littman DR (2016). The microbiota in adaptive immune homeostasis and disease. Nature.

[35] Atarashi K (2015). Th17 cell induction by adhesion of microbes to intestinal epithelial cells. Cell.

[36] Geuking MB (2011). Intestinal bacterial colonization induces mutualistic regulatory T cell responses. Immunity.

[37] Cheng WC (2016). Periodontitis-associated pathogens *P. gingivalis* and A. actinomycetemcomitans activate human CD14^+^ monocytes leading to enhanced Th17/IL-17 responses. Eur J Immunol.

[38] Zhang X (2015). The oral and gut microbiomes are perturbed in rheumatoid arthritis and partly normalized after treatment. Nat Med.

[39] Qin N (2014). Alterations of the human gut microbiome in liver cirrhosis. Nature.

[40] Blasco-Baque, V. *et al*. Periodontitis induced by Porphyromonas gingivalis drives periodontal microbiota dysbiosis and insulin resistance via an impaired adaptive immune response. Gut, gutjnl-2015-309897 (2016). 10.1136/gutjnl-2015-309897 PMC5531227 26838600

[41] Scher JU (2013). Expansion of intestinal Prevotella copri correlates with enhanced susceptibility to arthritis. Elife.

[42] Vaahtovuo J, Munukka E, Korkeamaki M, Luukkainen R, Toivanen P (2008). Fecal microbiota in early rheumatoid arthritis. J Rheumatol.

[43] Ley RE (2005). Obesity alters gut microbial ecology. Proc Natl Acad Sci USA.

[44] Chen J (2016). An expansion of rare lineage intestinal microbes characterizes rheumatoid arthritis. Genome Med.

[45] Caccese RG, Zimmerman JL, Carlson RP (1992). Bacterial lipopolysaccharide potentiates type II collagen-induced arthritis in mice. Mediators Inflamm.

[46] Sarkar S (2009). Regulation of pathogenic IL-17 responses in collagen-induced arthritis: roles of endogenous interferon-gamma and IL-4. Arthritis Res Ther.

[47] Yamazaki K (2007). Relationship of periodontal infection to serum antibody levels to periodontopathic bacteria and inflammatory markers in periodontitis patients with coronary heart disease. Clin Exp Immunol.

[48] Kato T (2014). Multiple omics uncovers host-gut microbial mutualism during prebiotic fructooligosaccharide supplementation. DNA Res.

[49] Kozich JJ, Westcott SL, Baxter NT, Highlander SK, Schloss PD (2013). Development of a dual-index sequencing strategy and curation pipeline for analyzing amplicon sequence data on the MiSeq Illumina sequencing platform. Appl Environ Microbiol.

[50] Caporaso JG (2010). QIIME allows analysis of high-throughput community sequencing data. Nat Methods.

[51] Edgar RC (2010). Search and clustering orders of magnitude faster than BLAST. Bioinformatics.

[52] Quast C (2013). The SILVA ribosomal RNA gene database project: improved data processing and web-based tools. Nucleic Acids Res.

[53] Takahashi N (2004). Selection of acid tolerant bifidobacteria and evidence for a low-pH-inducible acid tolerance response in Bifidobacterium longum. J Dairy Res.

[54] Noiri Y (2003). Effects of chlorhexidine, minocycline, and metronidazole on Porphyromonas gingivalis strain 381 in biofilms. J Periodontol.

[55] Yamamoto R (2011). Time course of gene expression during Porphyromonas gingivalis strain ATCC 33277 biofilm formation. Appl Environ Microbiol.

