# Supplementary material for: Aggravation of collagen-induced arthritis by orally administered Porphyromonas gingivalis through modulation of the gut microbiota and gut immune system
Source: Sci Rep. 2017 Jul 31;7:6955. doi: 10.1038/s41598-017-07196-7 (PMC5537233; doi:10.1038/s41598-017-07196-7)
Supplement: Supplementary file 1 — Supplementary information [file 41598_2017_7196_MOESM1_ESM.doc]

**Supplementary Information**

**Aggravation of collagen-induced arthritis by orally administered *Porphyromonas gingivalis* through modulation of the gut microbiota and gut immune system**

Keisuke Sato^1,2^, Naoki Takahashi^2,3^, Tamotsu Kato^4^, Yumi Matsuda^1,2^, Mai Yokoji^1,2^, Miki Yamada^1,2^, Takako Nakajima^5^, Naoki Kondo^6^, Naoto Endo^6^, Reiko Yamamoto^7**^, Yuichiro Noiri^8^, Hiroshi Ohno^4^, and Kazuhisa Yamazaki^1^*

^1^Research Unit for Oral-Systemic Connection, Division of Oral Science for Health Promotion, Niigata University Graduate School of Medical and Dental Sciences, Niigata, Japan

^2^Division of Periodontology, Niigata University Graduate School of Medical and Dental Sciences, Niigata, Japan

^3^Research Centre for Advanced Oral Science, Niigata University Graduate School of Medical and Dental Sciences, Niigata, Japan.

^4^Laboratory for Intestinal Ecosystem, RIKEN Centre for Integrative Medical Sciences (IMS), Yokohama, Japan

^5^Division of Dental Educational Research Development, Niigata University Graduate School of Medical and Dental Sciences, Niigata, Japan

^6^Division of Orthopedic Surgery, Department of Regenerative and Transplant Medicine, Niigata University Graduate School of Medical and Dental Sciences, Niigata, Japan

^7^Institute for Integrated Cell-Materials Science (WPI-iCeMS), Kyoto University, Sakyo, Kyoto, Japan.

**Present address: Laboratory for Integrative Omics, RIKEN Quantitative Biology Center (QBiC), Osaka, Japan.

^8^Division of Cariology, Operative Dentistry and Endodontics, Department of Oral Health Science, Niigata University Graduate School of Medical and Dental Sciences, Niigata, Japan

*Correspondence:

Prof Kazuhisa Yamazaki, E-mail: [kaz@dent.niigata-u.ac.jp](mailto:kaz@dent.niigata-u.ac.jp)

**Supplementary Figure S1. Experimental schedule of the study.**

Six-week-old DBA/1J mice were orally administered with *P. gingivalis* W83, *P. intermedia* ATCC 25611 (1 × 10^9^ CFU in 100 µl PBS with 2% carboxymethyl cellulose), or the vehicle only twice a week for 5 weeks. The next day after the final administration, mice were immunised with CII + CFA. Three weeks after primary immunisation, mice received a booster immunisation with CII + IFA. Samples were obtained at 3 weeks after booster immunisation.


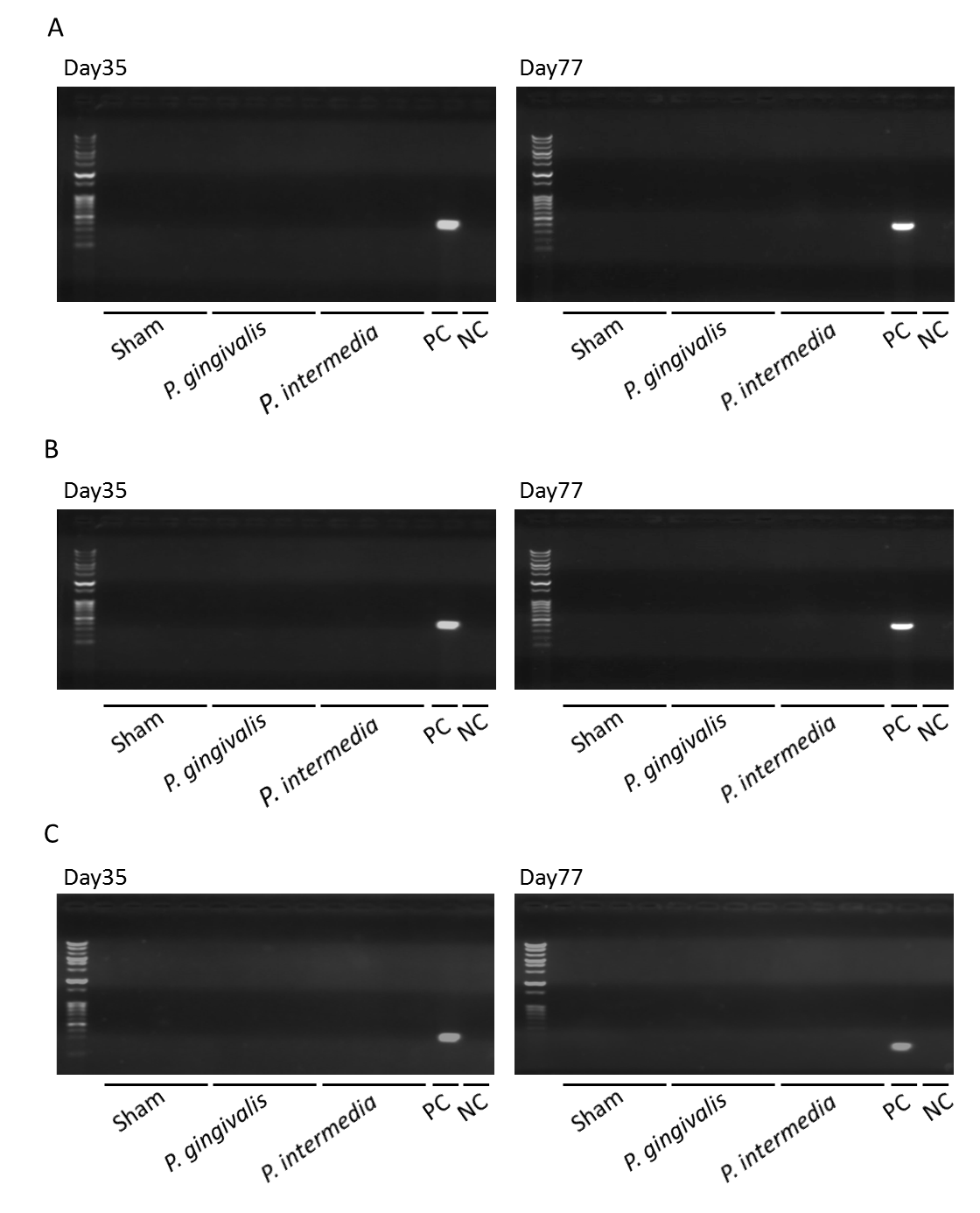


**Supplementary Figure S2. Detection of SFB, *P. gingivalis* and *P. intermedia* in faecal samples of *P. gingivalis*-, *P. intermedia*-, and sham-administered mice.**

(A) SFB-positive faecal samples were obtained from SFB-monoassociated gnotobiotic mice and used as a positive control.

(B) Genomic DNA of *P. gingivalis* W83 was used as a positive control.

(C) Genomic DNA of *P. intermedia* ATCC 25611 was used as a positive control.

Representative 1.2% agarose gels show the results of PCR amplification of DNA extracted from faecal samples of three mice in each group for detection of SFB, *P. gingivalis,* and *P. intermedia*. PC represents positive controls for respective bacterium. NC represents negative control containing no bacterial DNA.

None of these bacterial DNAs were detected in the samples.

**Supplementary Figure S3. Effect of P. gingivalis and P. intermedia administration on Th17 response in Peyer’s patch (A), inguinal lymph node (B), and spleen (C).**

The cells from each lymphoid tissue was stimulated with PMA/ionomycin and analysed by flow cytometry as described in the Materials and Methods.

Representative plots of one experiment with six mice per group are shown.

**Supplementary Materials and Methods**

**Detection of P. gingivalis and SFB in faeces**

The presence of specific 16S rRNA genes of SFB, P. gingivalis, and P. intermedia in faeces was detected by PCR. Bacterial genomic DNA was amplified using FastStart Essential DNA Green Master (Roche Molecular Systems, Inc., Pleasanton, CA) according to the manufacturer’s instructions. Primers for P. gingivalis, P. intermedia, SFB, and the universal 16S rRNA gene were as follows: P. gingivalis, 5′-AGGCAGCTTGCCATACTGCG-3′ and 5′-ACTGTTAGCAACTACCGATGT-3′; P. intermedia, 5′-CGTGGACCAAAGATTCATCGGTGGA-3′ and 5′-CCGCTTTACTCCCCAACAAA-3′; SFB, 5′-GACGCTGAGGCATGAGAGCAT-3′ and 5′-GACGGCACGGATTGTTATTCA-3′, and universal 16S rRNA, 5′-ACTCCTACGGGAGGCAGCAGT-3′ and 5′-ATTACCGCGGCTGCTGGC-3′.
